# Dopaminergic medication alters muscle synergy during sit-to-stand motion in Parkinson’s disease

**DOI:** 10.3389/fneur.2026.1753476

**Published:** 2026-03-23

**Authors:** Ken Kikuchi, Genko Oyama, Shingo Shimoda, Hiroyuki Hamada, Rukiye Aydin, Yoshihiro Kameyama, Kazunori Sato, Eriko Kitahara, Daiki Kamiyama, Atsushi Yamashita, Tomokazu Shimazu, Toshiyuki Fujiwara, Nobutaka Hattori, An Qi

**Affiliations:** 1 Department of Human and Engineered Environmental Studies, The University of Tokyo, Chiba, Japan; 2 Department of Neurology, Saitama Medical University, Saitama, Japan; 3 Department of Neurology, Juntendo University, Faculty of Medicine, Tokyo, Japan; 4 Graduate School of Medicine, Nagoya University, Nagoya, Japan; 5 Department of Rehabilitation, Juntendo University Hospital, Tokyo, Japan; 6 Department of Neurology, Saitama Neuropsychiatric Institute, Saitama, Japan; 7 Department of Rehabilitation Medicine, Juntendo University Graduate School of Medicine, Tokyo, Japan

**Keywords:** dopaminergic medication, kinematics, muscle synergy, neuromuscular coordination, Parkinson’s disease, sit-to-stand motion

## Abstract

**Background:**

Parkinson’s disease (PD) is a progressive neurodegenerative disorder that impairs motor function, thereby influencing daily activities, including sit-to-stand (STS) motion. Dopaminergic medication improves motor symptoms; however, its effects on neuromuscular control during STS motion remain unclear. This study investigated the effects of dopaminergic medication on muscle synergy and kinematic performance during STS motion in patients with PD.

**Methods:**

Fourteen patients with PD performed STS motion in the OFF and ON medication states. Surface EMG data from eight trunk and lower limb muscles and kinematic data of the center of mass (COM) trajectory were recorded. Muscle synergies were extracted using non-negative matrix factorization to assess temporal features and activation patterns. Kinematic features, including STS duration, time to seat-off, and COM displacement angle (initial to seat-off), were analyzed.

**Results:**

Dopaminergic medication significantly improved muscle synergy, achieving earlier initiation of the seat-off synergy and improved coordination between the propulsive and postural stabilization synergies. Neuromuscular improvement showed associations with changes in functional performance. Kinematic analysis revealed that the ON state was marked by shorter movement duration, reduced seat-off time, and a downward COM trajectory. These findings indicated that dopaminergic medication improves muscle synergy activation timing to enhance movement efficiency.

**Conclusion:**

These findings suggest that dopaminergic medication enhances the temporal precision of neuromuscular coordination and resolves the dysfunctional compensatory strategies during STS motion. These results provide novel insights into how dopamine modulates motor control in PD, with implications for clinical assessment and rehabilitation.

## 1 Introduction

Parkinson’s disease (PD), a progressive neurodegenerative disorder that impacts the motor system, is characterized predominantly by motor symptoms, including bradykinesia, rigidity, and resting tremors. The sit-to-stand (STS) motion is fundamental to daily living and ensuring functional independence. However, patients with PD often have difficulty performing STS motion because of an exaggerated hip flexion strategy and the impaired ability to smoothly switch between forward and upward body movements (1). Indeed, approximately 44% of patients with PD report these STS difficulties, which are strongly linked to independence and quality of life (2). Thus, understanding the mechanisms underlying impaired STS performance in patients with PD is clinically important.

STS motion is a complex task that can be functionally divided into two distinct phases: the flexion momentum phase, in which the body’s center of mass (COM) moves forward, and the subsequent transition phase, in which the body is lifted from the chair. Biomechanical studies have shown that STS motion in patients with PD is characterized by slower movements, particularly during flexion, (3, 4) which affects postural transitions and fall risk (5, 6). Despite these kinematic abnormalities, some studies have shown that surface electromyography (EMG) activity patterns during STS in patients with PD are comparable to those in healthy controls (7, 8). Additionally, patients with PD with abnormal STS performance, which was defined as a score of 2 or higher on Item 3.9 (“arising from chair”) of the Movement Disorder Society-Unified Parkinson’s Disease Rating Scale part III (MDS-UPDRS-III), complained of lower limb weakness, although this was not reflected in the activity of individual muscles such as the rectus femoris, vastus lateralis, vastus medialis, and vastus intermedius, gracilis, sartorius, gastrocnemius, and popliteus muscles (9). This previous study demonstrated that while a hip extension strength of less than 9 kg increased the risk of an abnormal STS task, this decreased muscle strength accounted for less than 25% of the variance in STS performance. These findings suggest that impaired STS performance in patients with PD may not stem from insufficient neural drive to individual muscles, but rather from the dysfunctional coordination of multiple muscles. While patients may experience functional weakness during the task, the fact that individual muscle activation levels remain comparable to those of healthy controls indicates that the primary deficit lies in the organization of muscle activity. Given the disconnection between individual muscle activation and kinematic performance, further investigation of the neuromuscular coordination mechanisms in individuals with PD is warranted.

Muscle synergy analysis is an ideal tool to address this topic, as this technique decomposes complex muscle activation patterns into coordinated modules, thus providing insight into the underlying organization of the central nervous system’s movement control (10–12). While our previous studies have identified stable muscle synergy modules during STS in elderly and patients with a stroke (13–16), the muscle synergy structure and dynamics during STS motion in PD remain poorly understood (17).

A comprehensive understanding of muscle activation patterns in patients with PD requires a consideration of each patient’s dopaminergic state. Levodopa and other drugs that modulate dopaminergic transmission are the most effective treatments for motor symptoms. These drugs improve motor control by suppressing pathological beta oscillations in the basal ganglia (18–20). Several studies have found no marked differences in muscle synergy structures between patients under medication (ON state) and those not being medicated (OFF state) during postural perturbation or gait (21, 22). However, others have found improved consistency in synergy organization during the ON state (23). Unlike postural perturbation and gait, which involve stable and cyclic movements, STS motion is a complex transitional task requiring the body to shift postural configurations from sitting to standing and accelerate upward against gravity, while controlling stability (1, 24, 25). As the core motor features of PD include akinesia and bradykinesia, which worsen in the OFF state (26, 27), this suggests that specific STS motion phases, particularly those requiring movement initiation and dynamic transition, may be differentially affected by the dopaminergic state.

Based on these findings, we hypothesized that muscle synergy expression would differ between the ON and OFF states during STS motion. Specifically, we predicted that, during the OFF state, muscle synergy patterns would reflect a compensatory strategy that prioritizes slow flexion and static stability. Conversely, we predicted that dopaminergic medication would improve the timing of functional modules, facilitating a more efficient transition between the seat-off and whole-body extension phases. In the present study, we combined kinematic and muscle synergy analyses to investigate these hypotheses and provide novel insights into how dopaminergic medication influences the modular control of movement in PD.

## 2 Methods

### 2.1 Participants

This study was conducted as part of a larger observational study aimed at exploring physiological biomarkers during OFF and ON medication testing using the MDS-UPDRS Part III with multiple sensor-based measurements. Measurements were conducted on 20 patients diagnosed with PD. The inclusion criteria were as follows: (1) a diagnosis of idiopathic PD according to the MDS Clinical Diagnostic Criteria (28) with a treatment regimen involving oral levodopa at least three times per day; (2) aged 18 years or older; (3) presence of wearing-off symptoms, defined as having a daily OFF time of 1 hour or more according to the MDS-UPDRS Part IV; (4) scheduled admission for a levodopa challenge test as part of routine clinical management; and (5) no dermatological conditions that would interfere with the placement of EMG electrodes. The exclusion criteria included: (1) inability to follow the study protocol; (2) a diagnosis of diabetes; (3) pregnancy; and (4) cognitive impairment, defined as a Mini-Mental State Examination (MMSE) score of less than 24. Six participants were excluded due to incomplete or unusable EMG and kinematic data, yielding a final sample size of 14 participants for analysis.

Fourteen patients with PD (mean age 61.36 ± 6.43 years, 10 males and 4 females) participated in this study; individual demographic and clinical data for each participant are provided in the Supplementary Table S1. During measurements, participants stood up from their preferred foot positions and rose independently from the chair. The MDS-UPDRS part III scores were 39.8 ± 11.4 (OFF state) and 12.4 ± 4.0 (ON state). In the OFF state, eight patients were Hoehn and Yahr stage II and six were stage III; in the ON state, 12 patients were stage II and two were stage III. The disease duration averaged 9.3 ± 2.9 years, the Barthel index was 90.0 ± 11.3, and the Levodopa Equivalent Daily Dose was 1452.3 ± 484.1 mg. Eleven patients exhibited dyskinesia in the ON state. All participants were asked to perform STS motion five times, although some completed only two trials in the OFF state due to mobility challenges. After excluding trials with signal noise, 62 and 68 trials in the OFF and ON states, respectively, were analyzed. All participants provided informed consent to participate and the study was approved by the Institutional Review Board of Juntendo University (Approval No. H21-0064-H01), in accordance with the Declaration of Helsinki and relevant ethical guidelines.

### 2.2 Experimental setting

The MDS-UPDRS III with multiple wearable devices and motion sensors was used during the levodopa challenge tests. In the levodopa challenge test, anti-Parkinsonian medication was withheld for at least 12 h prior to the morning of the assessment, followed by an intravenous infusion of levodopa at 1.5 × the usual morning dose administered over 30 min. To minimize the influence of transient or fluctuating effects of levodopa, ON-state assessments were initiated only after confirming that the participant had reached a stable peak-dose effect.

For STS (Item 3.9), as part of the MDS-UPDRS III, the participants performed STS motion five times. To standardize the starting conditions and eliminate momentum from previous repetitions, participants were instructed to maintain a static standing position for 2–3 s after each rise and then remain seated for at least 2–3 s between trials. In particular, during the ON state, researchers ensured that participants did not perform the movements rhythmically or prematurely. During the STS trials, participants were instructed to keep their arms crossed over their chests to minimize the contribution of upper-limb momentum to STS motion. Each trial was trimmed around the seat-off time to capture the full movement duration. The segment duration for each trial was first determined by visually inspecting synchronized video recordings to ensure that the entire movement from initial flexion to stable standing was captured. The segment length was then adjusted to account for individual variations in the timing of the pre- and post-seat-off phases. This ensured that both the initiation and transition to standing were fully included for each participant (see Supplementary Table S1). To compare data across trials with different movement speeds, dependent variables were time-normalized to a 100% STS cycle using spline interpolation. In this process, the start of STS motion was defined as the time when the horizontal COM displacement reached 5% of its total travel distance, while the end was defined as the time when the vertical COM displacement reached 95% of its total travel distance.

**Figure 1:**
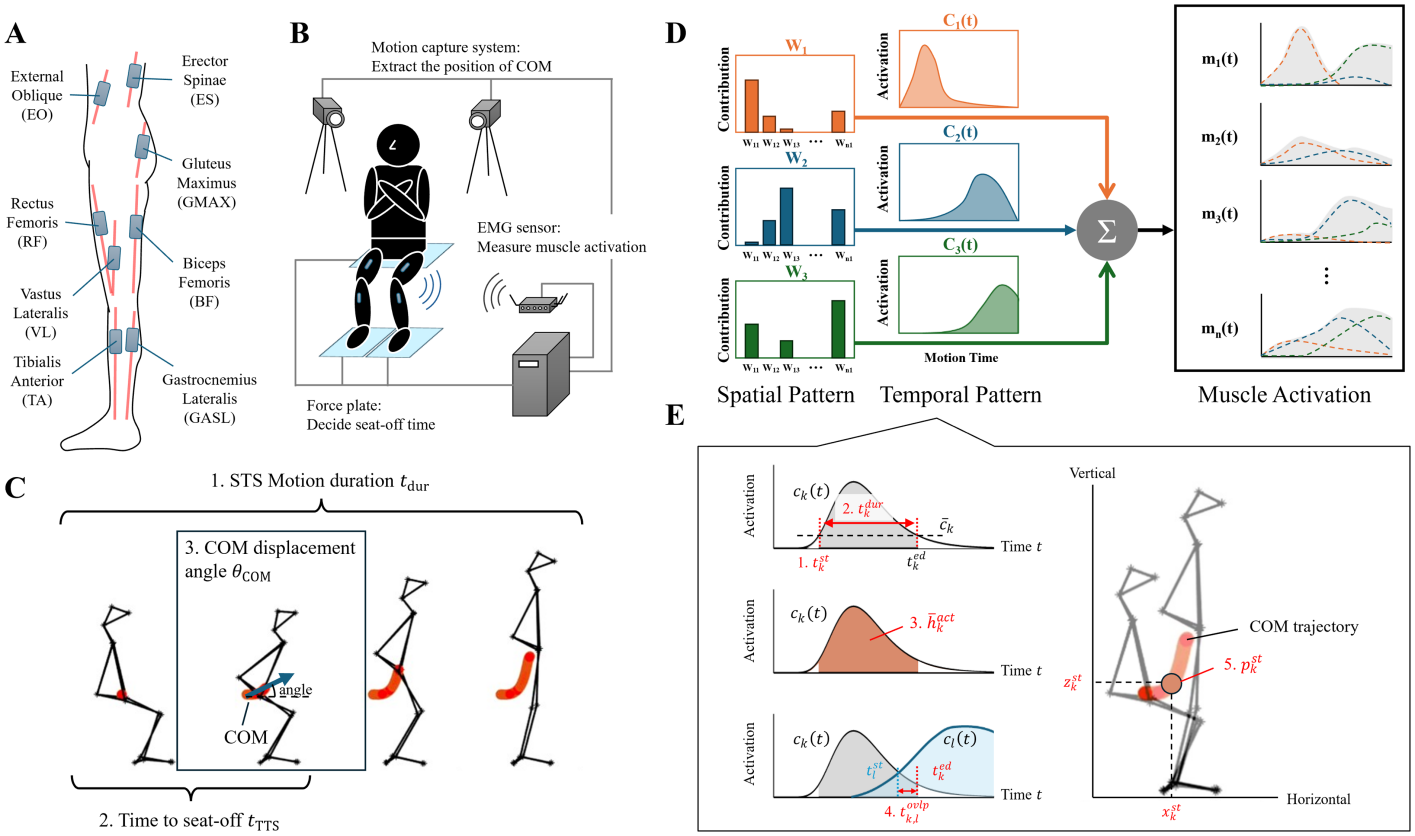
Experimental setup and data analysis. **(A)** Overview of the EMG electrode placement strategy for the eight trunk and lower limb muscles: the external oblique (EO), erector spinae (ES), gluteus maximus (GMAX), vastus lateralis (VL), rectus femoris (RF), biceps femoris long head (BFL), gastrocnemius lateralis (GASL), and tibialis anterior (TA). **(B)** Overview of the experimental setup, including the motion capture system, force plates, and EMG sensors. **(C)** The kinematic features used in the analysis, including STS motion duration, time to seat-off, and COM displacement angle (initial to seat-off). **(D)** Schematic of muscle synergy analysis using non-negative matrix factorization (NNMF). **(E)** Muscle synergy features extracted from the temporal pattern and COM position at synergy onset.

A motion capture system (OptiTrack, NaturalPoint Inc.), set at 100 Hz, and musculoskeletal modeling software (SIMM, Musculographics, Inc.) were applied to determine the COM using the Helen Heyes marker set with 26 markers on the participant’s body (29). The COM trajectories were low-pass filtered at a frequency of 6 Hz. Three force plates (TechGihan Corp.) were used to capture the ground reaction force data at 2,000 Hz, with the participants seated on one plate and their feet on the remaining two plates. The force data were low-pass filtered at 20 Hz, and used to identify the seat-off time when the vertical force decreased below 10 N. A wireless surface EMG device (Cometa Corp.) was applied to record the muscle activity at 2,000 Hz. Eight muscles were measured on both sides: the external oblique (EO), erector spinae (ES), gluteus maximus (GMAX), rectus femoris (RF), vastus lateralis (VL), biceps femoris long head (BFL), gastrocnemius lateralis (GASL), and tibialis anterior (TA) (Figure 1A). These EMGs electrodes were placed according to the SENIAM guidelines (30). EMG signals were band-pass filtered using a 4th-order Butterworth filter between 20 and 400 Hz, and then rectified and low-pass filtered at 4 Hz (31, 32). EMG signals were normalized based on the peak values in both the OFF and ON states. Figure 1B presents an overview of the experimental setup used in the study.

### 2.3 Data analysis

Kinematic performance was assessed using three key features (Figure 1C): STS motion duration ( $t_{\mathrm{dur}}$ ), time to seat-off ( $t_{\mathrm{TTS}}$ ), and COM displacement angle ( $\theta_{\mathrm{COM}}$ ), which were used to quantify the overall movement speed, timing of movement initiation, and trunk flexion strategy during STS motion, consistent with previous studies (4, 24).

To analyze and compare neuromuscular coordination in the OFF and ON states, muscle activation patterns were decomposed into a smaller number of muscle synergies using the non-negative matrix factorization (NNMF) to identify muscle synergies by separating the spatial and temporal patterns (Figure 1D) (33). Given the potential for shifts in neuromuscular strategies between pharmacological states, muscle synergies were extracted independently for the OFF and ON states of each participant using trial-by-trial data. This approach ensured that the distinct spatial and temporal characteristics of both the OFF and ON states were preserved and not confounded by the motor variability inherent in PD. Additionally, the EMG data from the eight muscles on each side were treated as independent inputs for the NNMF analysis. This approach ensured that the muscle synergies were extracted from the 8-muscle set of each limb independently, consistent with the unilateral framework described in previous research. The minimum number of synergies was determined as the minimum number required to achieve an overall $R^{2}>90\%$ , provided that the inclusion of an additional synergy did not increase the $R^{2}$ by more than 5%.

The temporal pattern features of the synergies were extracted to evaluate the differences between the OFF and ON states (Figure 1E). Muscle synergies were considered activated when the time-series activation exceeded the mean activation value for each trial. Temporal features were extracted from each muscle synergy, including the start time, duration, average activation value, and overlap time between every pair of muscle synergies (14–16). Additionally, to investigate how the STS motion strategies differed between states, we analyzed the horizontal and vertical positions of the COM at the onset of synergy activation. For a more detailed explanation of these analytical methods, please refer to the Supplemental materials.

### 2.4 Statistical analysis

To maximize statistical power and account for both inter-individual and trial-to-trial variability, linear mixed models (LMMs) were applied to analyze kinematic and muscle synergy features. This hierarchical approach leverages the full trial-by-trial dataset while treating participants as random effects, providing robust estimates even in clinical cohorts with limited sample sizes. For the kinematic features, the fixed effect was the state (OFF *vs.* ON), whereas for the muscle synergy features, the fixed effects included the state and synergy number. When analyzing the overlap time, the synergy transitions (from 1 to 2 and from 2 to 3) were used as fixed effects instead of the synergy numbers. Although the lateral factor was not included as a fixed effect, muscle synergy features from both legs were included in the analysis because our primary focus was on the effects of dopaminergic medication on the forward and vertical components of STS motion.

Model assumptions were verified using diagnostic plots (Supplementary Figure S1). For the Average Activation Value, which satisfied model assumptions, significance was determined using *F*-statistics with the Satterthwaite approximation. For dependent variables that exhibited heteroscedasticity characterized by an upward trend in Scale-Location (S-L) plots such as start time, duration, and COM horizontal position, square root transformations were applied to stabilize the variance. Furthermore, to ensure robustness against potential violations of normality and homoscedasticity, a parametric bootstrap method (1,000 iterations) was consistently employed. Significance for the models was determined using chi-square statistics derived from the likelihood ratio test framework.

When a significant interaction between the state and synergy was observed, pairwise comparisons were applied to examine the differences between the OFF and ON states for each muscle synergy. Regarding the COM horizontal and vertical positions at the onset of synergy activation, pairwise comparisons were also applied to examine the differences between the muscle synergies in the OFF and ON conditions. The estimated marginal means from the model were used for comparisons, with multiple comparisons adjusted using the Holm correction method. All statistical analyses were conducted using JASP 0.18.3 (34). The alpha level was set at *p* < 0.05.

## 3 Results

### 3.1 The number of muscle synergies

**Figure 2:**
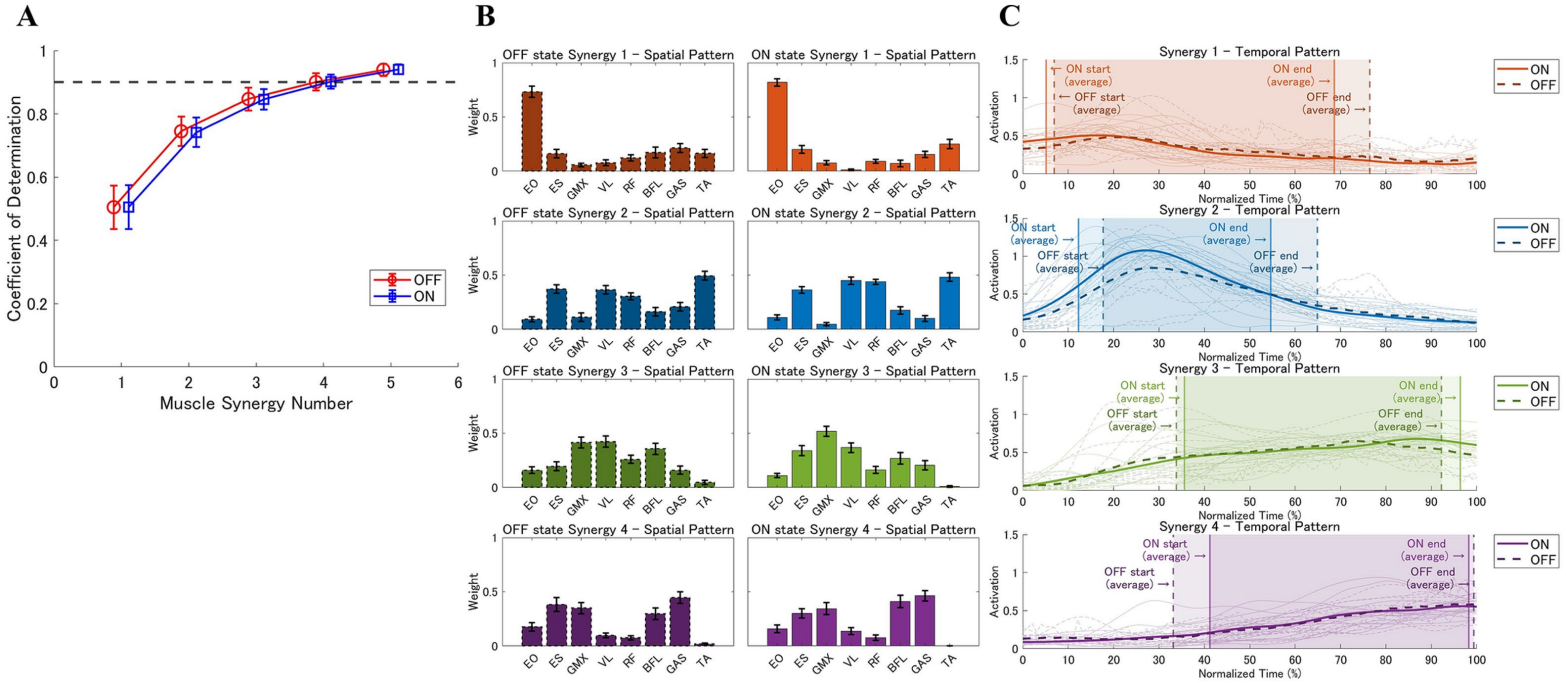
Muscle synergies. **(A)** Coefficients of determination (*R*^2^) for the different numbers of muscle synergies in the OFF and ON states. Four synergies were selected as the optimal number. **(B)** The muscle weights of the four identified muscle synergies. The left column shows the spatial pattern in the OFF state, and the right column shows the spatial pattern in the ON state. Each synergy represents a distinct muscle spatial coordination pattern. **(C)** Temporal activation patterns of the four muscle synergies; dashed and solid lines represent the mean activation patterns for the OFF and ON states, respectively. Vertical lines denote the average onset and end times for each synergy. The shaded areas bounded by vertical lines indicate the timing and duration of muscle synergy activation. This figure illustrates how dopaminergic medications influence the timing and duration of muscle synergy activation.

Figure 2A presents the coefficients of determination $R^{2}$ for the different muscle synergy numbers. With four muscle synergies, the $R^{2}$ was 90.07 ± 2.73% in the OFF state and 90.19 ± 2.21% in the ON state. While the use of five synergies slightly increased the $R^{2}$ (OFF: 93.95 ± 1.92%; ON: 94.09 ± 1.56%), the incremental increase remained less than 5%. These results indicate that a four-synergy model is sufficient to represent most of the muscle activation during STS motion in patients with PD, providing an optimal balance between reconstruction accuracy and physiological interpretability.

Figures 2B,C present the spatial and temporal patterns of the four muscle synergies, respectively. Based on muscle weight and activation timing, synergy 1 initiated forward movement by bending the trunk; synergy 2 played a role of seat-off through tilting the shank forward and elevating the hip; synergy 3 controlled whole-body extension through hip and knee extension; and synergy 4 managed postural stabilization.

### 3.2 Temporal features of muscle synergy

**Figure 3:**
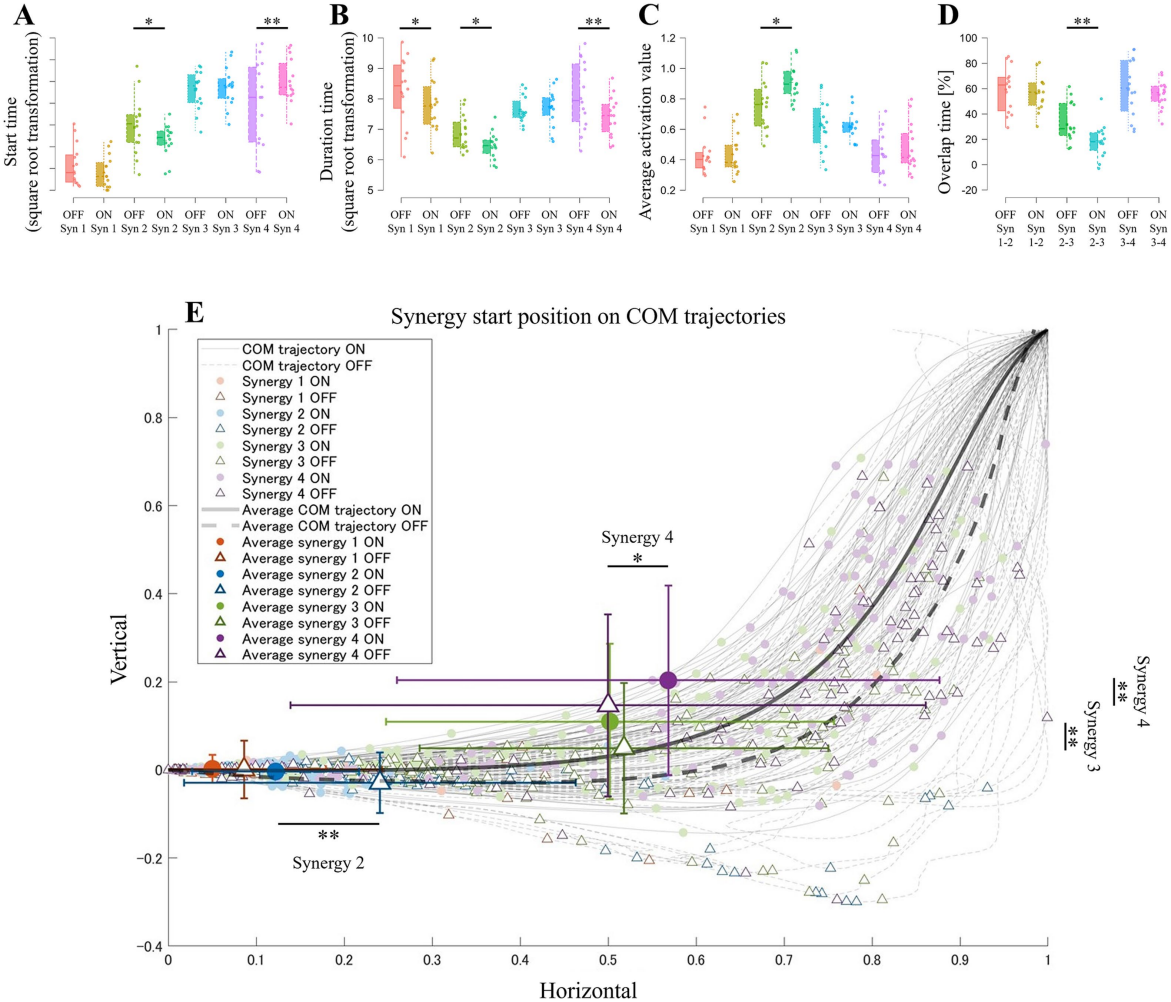
Comparison of the muscle synergy features between the OFF and ON states for each synergy. **(A)** The mean start time and **(B)** mean duration of each muscle synergy. **(C)** The mean activation value for each synergy. **(D)** The mean overlap time between synergies. **(E)** The mean horizontal and vertical COM position at the onset of each synergy. *p < 0.05. **p < 0.01.

LMMs showed significant main effects of state, synergy, and their interactions on the temporal features of muscle synergy (Table 1 and Supplementary Table S2). The start and duration of the state and the synergy interactions were identified, and pairwise comparisons revealed differences between states in Synergy 2 (OFF > ON, *p* = 0.015) and Synergy 4 (OFF < ON, *p* < 0.001) for the start time (Figure 3A) and in Synergy 1 (OFF > ON, *p* = 0.021), Synergy 2 (OFF > ON, *p* < 0.028), Synergy 4 (OFF > ON, *p* < 0.001) for the duration time (Figure 3B). Figure 2C shows the average start and end transitions between the OFF and ON states, indicating a delayed muscle activity timing for seat-off in the OFF state. The state and synergy interactions were also observed for the average activation values. Synergies 2 (OFF < ON, *p* < 0.001) were significant for average activation (Figure 3C), indicating lower muscle activity during seat-off in the OFF state, whereas the ON state efficiently activated the muscles for seat-off. For overlap time (Figure 3D), pairwise comparisons showed significance in synergies 2–3 (OFF > ON, *p* < 0.001). Given the extended duration of Synergy 2 in the OFF state and the lack of notable differences in the start time and duration time between the OFF and ON states of Synergy 3, the increased overlap in OFF state indicates prolonged muscle group activity during seat-off. When examining the position of the COM at the onset of synergy, both the horizontal and vertical of the state and the synergy interactions were observed (Figure 3E). Regarding the horizontal position, the COM position in the OFF state for Synergy 2 was significantly more forward than that in the ON state (OFF > ON, *p* < 0.001). This indicates that synergy activation occurred during the more forward COM shift. For Synergy 4, the COM position in the OFF state was significantly lower than that in the ON state (OFF < ON, *p* = 0.017). Additionally, in the comparison of adjacent synergies within each state, the COM positions for synergies 3 and 4 in the OFF state differed from their order (Synergy 1 < 2 < 3 > 4; see in Supplementary Figure S2A). These findings indicate that the Synergy 4 activation in OFF state begins earlier in the horizontal direction. Regarding the vertical position, significant changes were observed in Synergies 3 and 4. For Synergy 3, the COM height in the OFF state was significantly lower than that in the ON state (OFF < ON, *p* < 0.001), indicating that extension activation occurred during deeper forward bending. Conversely, in Synergy 4, the COM height in the OFF state was also significantly lower (OFF < ON, *p* < 0.001), indicating that vertical activation occurred at an early stage. In Synergy 2, vertical differences were minor, with notable differences being predominantly horizontal.

**Table 1:** Linear mixed model (LMM) analysis of the temporal features of muscle synergies.

Muscle synergy features (method)	Fixed effect	Estimate	SE	t	*p*-value	Random effect (SD)	Residual (SD)
Start time (square root transformation + parametric bootstrap LMM)	Intercept	4.200	0.124	33.908	< 0.001	0.420	1.686
	State (1)	−0.020	0.053	−0.379	0.705		
	Synergy (1)	−2.231	0.091	−24.616	< 0.001		
	Synergy (2)	−0.607	0.091	−6.697	< 0.001		
	Synergy (3)	1.481	0.091	16.334	< 0.001		
	State (1) ✻ synergy (1)	0.167	0.091	1.837	0.066		
	State (1) ✻ synergy (2)	0.315	0.091	3.471	< 0.001		
	State (1) ✻ synergy (3)	−0.041	0.091	−0.45	0.653		
Duration time (square root transformation + parametric bootstrap LMM)	Intercept	7.516	0.087	85.942	< 0.001	0.295	1.216
	State (1)	0.163	0.038	4.305	< 0.001		
	Synergy (1)	0.502	0.065	7.678	< 0.001		
	Synergy (2)	−0.870	0.065	−13.298	< 0.001		
	Synergy (3)	0.155	0.065	2.377	0.018		
	State (1) ✻ synergy (1)	0.040	0.065	0.615	0.538		
	State (1) ✻ synergy (2)	0.022	0.065	0.342	0.732		
	State (1) ✻ synergy (3)	−0.209	0.065	−3.190	0.001		
Average activation value (standard LMM)	Intercept	0.590	0.012	49.433	< 0.001	0.037	0.214
	State (1)	−0.022	0.007	−3.358	< 0.001		
	Synergy (1)	−0.159	0.012	−13.786	< 0.001		
	Synergy (2)	0.249	0.012	21.587	< 0.001		
	Synergy (3)	0.039	0.012	3.403	< 0.001		
	State (1) ✻ synergy (1)	0.016	0.012	1.417	0.157		
	State (1) ✻ synergy (2)	−0.048	0.012	−4.2	< 0.001		
	State (1) ✻ synergy (3)	0.028	0.012	2.404	0.016		
Overlap time (parametric bootstrap LMM)	Intercept	46.865	1.804	25.978	< 0.001	5.928	23.985
	State (1)	3.384	0.864	3.916	< 0.001		
	Synergy (1)	10.987	1.216	9.037	< 0.001		
	Synergy (2)	−21.544	1.216	−17.719	< 0.001		
	State (1) ✻ synergy (1)	−1.858	1.216	−1.528	0.127		
	State (1) ✻ synergy (2)	2.963	1.216	2.437	0.015		
COM horizontal position (square root transformation + parametric bootstrap LMM)	Intercept	0.465	0.016	29.526	< 0.001	0.052	0.233
	State (1)	0.015	0.007	2.038	0.042		
	Synergy (1)	−0.316	0.013	−25.211	< 0.001		
	Synergy (2)	−0.092	0.013	−7.356	< 0.001		
	Synergy (3)	0.221	0.013	17.659	< 0.001		
	State (1) ✻ synergy (1)	0.018	0.013	1.401	0.161		
	State (1) ✻ synergy (2)	0.043	0.013	3.462	< 0.001		
	State (1) ✻ synergy (3)	−0.004	0.013	−0.356	0.722		
COM vertical position (parametric bootstrap LMM)	Intercept	0.058	0.011	5.524	< 0.001	0.036	0.133
	State (1)	−0.020	0.004	−4.803	< 0.001		
	Synergy (1)	−0.058	0.007	−8.160	< 0.001		
	Synergy (2)	−0.076	0.007	−10.693	< 0.001		
	Synergy (3)	0.020	0.007	2.757	0.006		
	State (1) ✻ synergy (1)	0.017	0.007	2.414	0.016		
	State (1) ✻ synergy (2)	0.005	0.007	0.721	0.471		
	State (1) ✻ synergy (3)	−0.012	0.007	−1.687	0.092		

The optimal method was selected for all variables based on diagnostic plots. Specifically, the standard LMM was used for variables where homogeneity of variance was maintained (Average activation value); the results from the parametric bootstrap method were reported for variables where transformation was difficult or homogeneity of variance was insufficient (Overlap time and COM vertical position); and the parametric bootstrap LMM method after square root transformation was used for variables requiring transformation (Start time, Duration time and COM horizontal position).

### 3.3 Kinematic features

The LMMs revealed significant differences between the ON and OFF states for all the kinematic features (Supplementary Figure S3). The STS motion duration was significantly increased in the OFF state compared to that in the ON state (estimate = 0.96, SE = 0.19, *t* = 5.18, *p* < 0.001). Specifically, the mean difference in STS motion duration was 1.92 s (95% CI [1.19, 2.65]). Further, the time to seat-off was longer in the OFF state (estimate = 0.47, SE = 0.10, *t* = 4.90, *p* < 0.001). The COM displacement angle (initial to seat-off) showed a downward trajectory in the OFF state (estimate = − 3.59, SE = 0.49, *t* = − 7.34, *p* < 0.001).

## 4 Discussion

Overall, this study examined the effects of dopaminergic medication on the kinematic performance and neuromuscular organization during STS motion in patients with PD. Although prior research has extensively documented kinematic abnormalities in STS motion in patients with PD, the specific impact of dopaminergic medication on the underlying neuromuscular control remains to be fully elucidated. Our kinematic analyses corroborated the findings of prior studies (8, 35), demonstrating that the OFF state was characterized by less efficient performance, including a prolonged time to seat-off and a downward COM trajectory. Consistently, our analysis revealed that temporal muscle Synergy 2, which plays a crucial role of seat-off in the preparatory and initiation phases of movement, exhibited a delayed onset in the OFF state compared with the ON state, both in terms of temporal parameters and spatial COM position.

The delayed onset of the muscle synergy for seat-off likely underlies the kinematic abnormalities observed. The prolonged time to seat-off observed in the OFF state is thought to result from inappropriate peak torques and extended torque generation times in the hip and ankle joints (4, 24). These findings suggest that improvements in STS performance with dopaminergic medication are not merely due to a general increase in movement speed. Further, our results indicate that dopaminergic medication directly addresses the dopaminergic dysfunction of the basal ganglia to improve the timing of the muscle synergy activation required to initiate STS motion. This mechanism is consistent with reports that levodopa partially restores the excitability of the primary motor cortex (M1), thereby preparing an appropriate output for voluntary movements (20). Furthermore, the direct impact of levodopa treatment on the overall improvement in motor control likely enhances the quality of preparatory postural strategies (36). This suggests that dopaminergic medication contributes to the alleviation of akinesia symptoms observed in STS motion, which is consistent with other reports that it can enhance the speed and amplitude of muscle activity and ameliorate abnormal muscle activity patterns (19).

Our muscle synergy results for the start time and duration time indicated that the muscle synergy for postural stabilization (Synergy 4) was activated earlier in the OFF state. A deeper analysis of the COM trajectory at synergy onset further clarified the mechanisms behind these coordination deficits. In the OFF state, COM trajectory at synergy onset of began at more backward and lower positions compared to the ON state. These findings indicate that in the OFF state, muscle synergies are recruited while the body is in a kinematically disadvantaged and inefficient posture, necessitating excessive co-activation to maintain balance. These findings support a compensatory strategy in which postural control appears to prioritize static stability in the OFF state (37, 38).

These delayed or early activations of muscle synergies may result in co-contraction, which interferes with the preceding or subsequent synergy. Indeed, coactivation of agonists and antagonists was identified in the quadriceps and hamstring muscles during postural perturbation, as well as in the dorsiflexors and soleus muscles, following the initiation of voluntary ankle dorsiflexion in patients with PD (39, 40). These temporal and dynamic alterations in synergy activation may further reflect an impaired central motor control, including deficits in switching movement direction, such as bradykinesia symptoms observed in STS motion, exacerbated by the absence of dopaminergic inputs (24, 41, 42). Dopaminergic medication likely improves these deficits by improving the timing of competing synergies, thereby facilitating a more efficient transition between seat-off and whole-body extension (23, 43). In the OFF state, the impaired temporal coordination between these two phases may necessitate the premature activation of the stabilizing synergy (Synergy 4) to compensate for kinematic instability. By improving this transition, medication reduces the need for such early compensatory stabilization, allowing for a more efficient movement pattern. The restoration of precise temporal coordination through dopaminergic medication not only explains the observed kinematic improvements, but also highlights the direct effect on central motor control in patients with PD.

Additionally, it should be noted that the levodopa challenge test typically employs a levodopa dose 1.5 times the patient’s optimal morning dose to elicit the maximum pharmacological response. Doses exceeding this threshold may induce transient effects such as levodopa-induced dyskinesia but often produce more pronounced clinical improvement than usual doses (44). In this study, while 11 of 14 participants exhibited dyskinesia at the peak-dose stage (see the Supplementary Table S1), the fact that stable COM control and appropriate temporal characteristics of muscle synergies (i.e., smooth COM displacement and synergy activation timing) were achieved even under such potent transient pharmacological effects indicates that dopaminergic agents allow the underlying motor program to function appropriately.

These findings could have substantial implications for clinical assessment and rehabilitation strategies for patients with PD. Firstly, while the characteristics of STS motion in patients with PD in the OFF state have been frequently observed from initial trunk flexion through seat-off (8, 35), earlier activation of the muscle groups involved in postural stabilization was also observed in our study. Considering that patients with mild PD use a hip flexion strategy that includes the need for greater postural stability during the seat-off phase, (38) measuring the EMG of the trunk and lower limb muscles involved in postural stabilization during STS motion, such as the ES, GMAX, BFL, and GASL, may facilitate the early detection of the OFF state. Furthermore, the identified deficits in the temporal precision of muscle synergies and the inefficient COM trajectory in the OFF state suggest that interventions should focus on reestablishing the precise temporal coordination of muscle synergies and optimizing the preparatory COM trajectory for efficient STS initiation. For example, our results indicate that interventions should target the suppression of premature Synergy 4 activation and the promotion of properly timed Synergy 2 activation. This could be achieved through real-time biofeedback of the COM trajectory and muscle activity during the initial STS phase, thereby guiding patients to activate the propulsion synergy (Synergy 3) only after reaching a kinematically advantageous position. This approach further aligns with findings showing that real-time biofeedback on trunk tilt during walking produces immediate effects, such as reduced trunk tilt, stride length, and walking speed (45), and that task-specific training using external audiovisual stimuli improves STS speed and efficiency (46, 47). Furthermore, functional electrical stimulation (FES) is an effective method for prompting activity at the appropriate timing for each synergy, and it has already been shown to be effective in improving gait and bradykinesia (48). Therefore, the present study provides a novel synergy-based framework for developing such targeted interventions.

This study has some limitations. First, the sample size was relatively small (*n* = 14), and *a priori* power analysis was not performed. However, using LMMs allowed us to leverage the full trial-by-trial dataset, ensuring robust statistical estimates. The highly significant differences observed in temporal synergy parameters (e.g., *p* < 0.001) suggest that dopaminergic medication substantially affects motor coordination, even within this cohort. Nevertheless, these findings require confirmation in larger populations with different PD subtypes (49). Second, we focused only on the trunk and lower limb muscles, whereas the upper limb muscles may have affected the STS performance. The upper limbs often provide essential support and stability. Excluding upper-limb analysis from this study limits fully capturing the holistic motor strategies that patients with PD use in real-world scenarios. Future research incorporating upper-limb muscle synergies and their interaction with the trunk and lower limbs will be essential to provide a more comprehensive understanding of STS strategies in patients with PD. Third, we did not assess the cognitive factors affecting movement control in the ON and OFF states. Future research is needed to combine muscle synergy analysis with neuroimaging could clarify the neural mechanisms underlying these changes (50). Finally, longitudinal studies may help assess how synergistic modulation evolves over time.

## 5 Conclusion

Overall, the results of this study provide novel insights into the modulation of neuromuscular control of functional movements by dopaminergic therapy, particularly during STS motion, in patients with PD. The compromised central motor control in the OFF state is further reflected in the impaired amplitude and timing of muscle synergy activation, leading to challenges in initiating movements and transitioning between phases. These difficulties may be indicative of core PD symptoms such as akinesia and bradykinesia. By integrating kinematic and synergy-based analyses, the present study showed that dopaminergic therapy not only enhances overall STS performance but also improves the temporal precision of muscle synergies. Specifically, it appears to facilitate a more efficient functional transition between seat-off and whole-body extension, addressing the excessive temporal overlap and the resulting need for early compensatory stabilization observed in the OFF state. These findings have significant implications for the clinical evaluation and rehabilitation strategies of patients with PD, suggesting that interventions should focus on optimizing the temporal coordination of muscle synergies and preparatory postural strategies in patients with PD.

## Data Availability

The raw data supporting the conclusions of this article will be made available by the authors, without undue reservation.
