# Supplementary material for: Dopaminergic medication alters muscle synergy during sit-to-stand motion in Parkinson’s disease
Source: Front Neurol. 2026 Mar 23;17:1753476. doi: 10.3389/fneur.2026.1753476 (PMC13051405; doi:10.3389/fneur.2026.1753476)
Supplement: Supplementary file 1 [file Data_Sheet_1.pdf]

Supplementary materials

Supplemental material

Data Analysis

Kinematics features

To quantitatively investigate the effects of dopaminergic medication on STS motion by analyzing the kinematic performance, representative kinematic metrics were determined using the COM data, based on the methods of prior studies. The start and end of the STS motion were defined based on the COM position. The start and end frames were defined as the times at which 5% (COM_x) and 95% (COM_z) of the total distance traveled by the COM in the horizontal and vertical direction was achieved (t_{st} and t_{ed}), respectively. This study focused on three features of motion kinematics (Figure 1C):

1. STS motion duration t_{dur}: A determination of the STS motion duration involving measurement of the interval between the start and end of the STS motion, determined as follows: $t_{dur} = t_{ed} - t_{st}$.
2. Time to seat-off t_{TTS}: A measurement of the duration from the start of the STS motion to seat-off as an indicator of the delay in motion initiation, determined as follows: $t_{TTS} = t_{seat-off} - t_{st}$.
3. COM forward flexion angle θ_{COM}: The angle to the horizontal calculated from the COM

position at the start of the STS motion and at the time of seat-off, as a metric to evaluate the initial STS motion strategy with deeper forward bending, determined as follows: $\theta_{COM} = arctan\left(\frac{COM_z(t_{seat-off})-COM_z(t_1)}{COM_x(t_{seat-off})-COM_x(t_1)}\right)$.

Muscle synergy model

Human STS motion is characterized by multi-joint movements, facilitated by muscle coordination. Within the framework of the muscle synergy model, muscle activation is represented as a linear summation of the spatiotemporal patterns, as expressed mathematically in Equation (2).

$$M = WC, \quad (2)$$

where the matrices M, W, and C denote the muscle activation, spatial pattern, and temporal pattern matrices, respectively. Matrix M comprises the muscle activation vectors m_i (i = 1, 2, …, n) representing the activation levels of n distinct muscles.

Figure 1D presents a schematic of the muscle synergy model, which uses three muscle synergies to represent n muscle activations with spatial and temporal patterns. The spatial patterns ($w_{1, 2, 3}$) indicate the contribution of each muscle to each synergy, whereas the temporal patterns ($c_{1, 2, 3}$) represent the synergy activation timing. Non-negative matrix factorization (NNMF) was employed to compute matrices W and C for each trial of each participant. The muscle synergy orders were organized according to the spatial and temporal patterns, as established in previous

STS motion studies. The coefficient of determination, R^2, was computed for various numbers of muscle synergies. The maximum number of iterations of the NNMF function was set to a default value of 100. To ensure the robustness of the results, the calculation was repeated 150 times for each leg with different random initial values, while the solution with the highest coefficient of determination, R^2, was selected. The number of muscle synergies that adequately represented muscle activation was determined. This study adopted the minimum number of synergies to achieve a coefficient of determination of 90% to ensure accurate reconstruction of muscle activity through OFF/ON conditions.

Muscle synergy features

Temporal features were calculated to examine the differences in temporal patterns between the OFF and ON states. These temporal features were selected to describe the timing and amplitude patterns (Figure 1E). The start time, duration, average activation value, and overlap time were calculated based on previously described methods. In addition, to investigate how the STS motion strategies differed between the OFF and ON states, the COM position at the onset of synergy activation was examined. The horizontal ($\widetilde{COM}_x$) and vertical ($\widetilde{COM}_z$) COM trajectories were spatially normalized using minimum–max normalization (scaled between 0 and 1 based on individual minimum and maximum values). The spatially normalized COM position is denoted by a tilde (i.e., ~) throughout this manuscript. The *k*-th muscle synergy was considered activated

when the timing of activation exceeded the mean activation $\bar{c}_k$ during each trial. To compare the trials of varying durations, the time axis was normalized to 0-100% based on the COM-defined start time (t_{st}) to the end time (t_{ed}) of the STS motion, as defined in the kinematics feature section. Temporally normalized time is denoted by a tilde (i.e., ~). After temporal normalization, the selected temporal features were extracted as follows:

1. Start time $\tilde{t}_{st,k}$: the initial activation time of the *k*-th muscle synergy, determined as follows: $\tilde{t}_{st,k} = arg\min_t c_k(t) > \bar{c}_k$.
2. Duration time $\tilde{t}_{dur,k}$: the interval between the start time $\tilde{t}_{st,k}$ and end time $\tilde{t}_{ed,k}$, calculated as follows: $\tilde{t}_{ed,k} = arg\max_t c_k(t) > \bar{c}_k$, $\tilde{t}_{dur,k} = \tilde{t}_{ed,k} - \tilde{t}_{st,k}$.
3. Average activation value $\bar{h}_{act,k}$: the mean activation value of the muscle synergy between the start $\tilde{t}_{st,k}$ and end time $\tilde{t}_{ed,k}$, calculated as follows: $\bar{h}_{act,k} = \frac{1}{\tilde{t}_{dur,k}} \int_{\tilde{t}_{st,k}}^{\tilde{t}_{ed,k}} c_k(t)dt$.
4. Overlap time $\tilde{t}_{ovlp,k,l}$: the overlap time between every two muscle synergies *k* and *l*, determined as follows: $\tilde{t}_{ovlp,k,l} = \tilde{t}_{ed,k} - \tilde{t}_{st,l}$.
5. COM horizontal and vertical positions at the onset of synergy activation $p_{st,k}$: the horizontal ($x_{st,k}$) and vertical ($z_{st,k}$) positions of the COM at the initial activation time of the *k*-th muscle synergy, determined as follows: $p_{st,k} = \left(x_{st,k}, z_{st,k}\right) = \left(\widetilde{COM}_x(\tilde{t}_{st,k}), \widetilde{COM}_z(\tilde{t}_{st,k})\right)$.

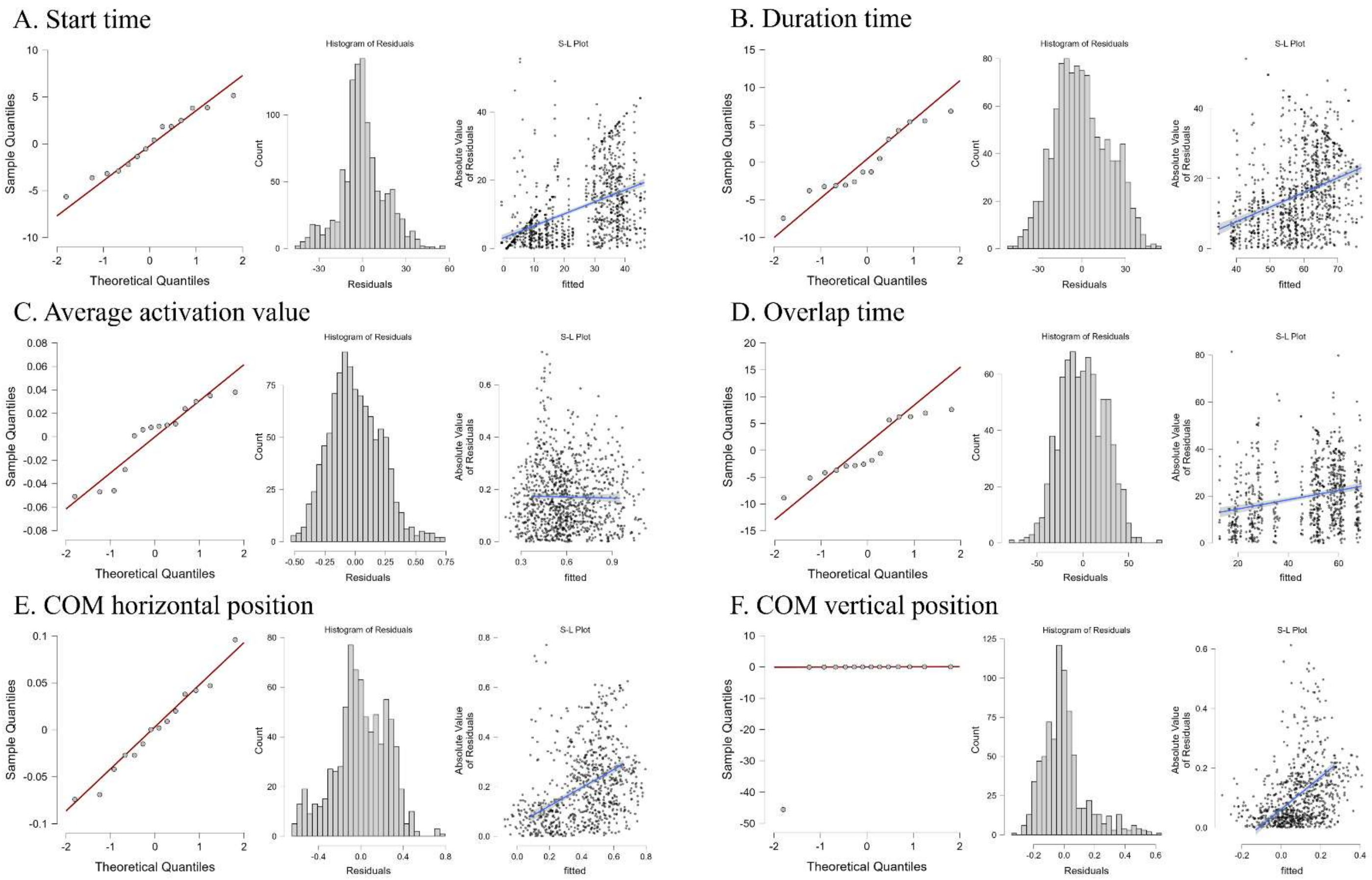

Figure S1: Model diagnostic plots for all dependent variables. For each of the six variables, three diagnostic plots are provided: (1) Q-Q plots of random intercepts to verify the normality of participant-level random effects; (2) histograms of residuals to assess the normality of the error terms; and (3) Scale-Location (S-L) plots to evaluate homoscedasticity. These visualizations informed the selection of appropriate statistical treatments, such as data transformation or parametric bootstrapping, to ensure the robustness of the linear mixed model (LMM) results.

Supplementary materials

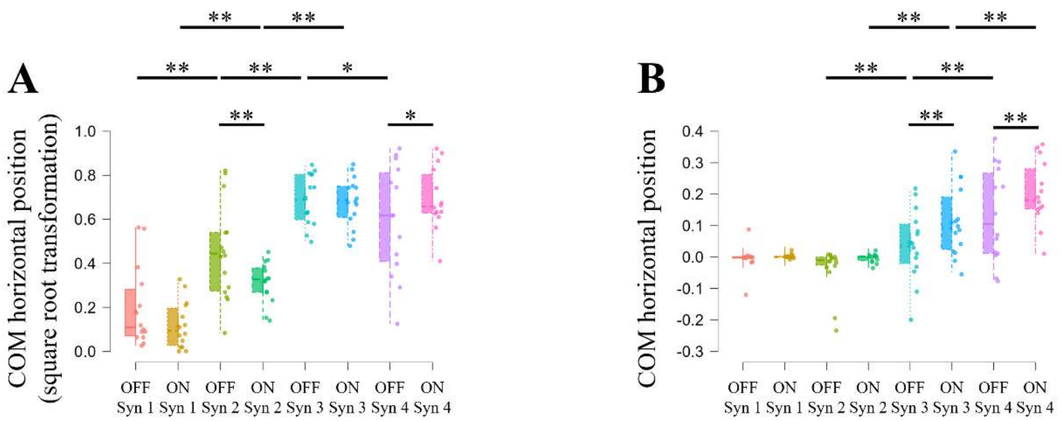

Estimated marginal means

				95% CI	
tate	synergy	Estimate	SE	Lower	Upper
OFF	1	0.182	0.025	0.132	0.231
ON	1	0.117	0.024	0.069	0.165
OFF	2	0.431	0.025	0.382	0.480
ON	2	0.315	0.024	0.267	0.362
OFF	3	0.697	0.025	0.647	0.746
ON	3	0.676	0.024	0.628	0.724
OFF	4	0.610	0.025	0.561	0.660
ON	4	0.693	0.024	0.646	0.741

Estimated marginal means

				95% CI	
state	synergy	Estimate	SE	Lower	Upper
OFF	1	-0.003	0.015	-0.033	0.028
ON	1	0.003	0.015	-0.027	0.032
OFF	2	-0.033	0.015	-0.063	-0.003
ON	2	-0.003	0.015	-0.033	0.026
OFF	3	0.046	0.015	0.016	0.076
ON	3	0.11	0.015	0.081	0.139
OFF	4	0.143	0.015	0.113	0.173
ON	4	0.204	0.015	0.174	0.233

Figure S2: Comparison of muscle synergy features between the synergies within each state. Mean (A) horizontal and (B) vertical COM positions at the onset of each synergy. $*p < 0.05$, $**p < 0.01$.

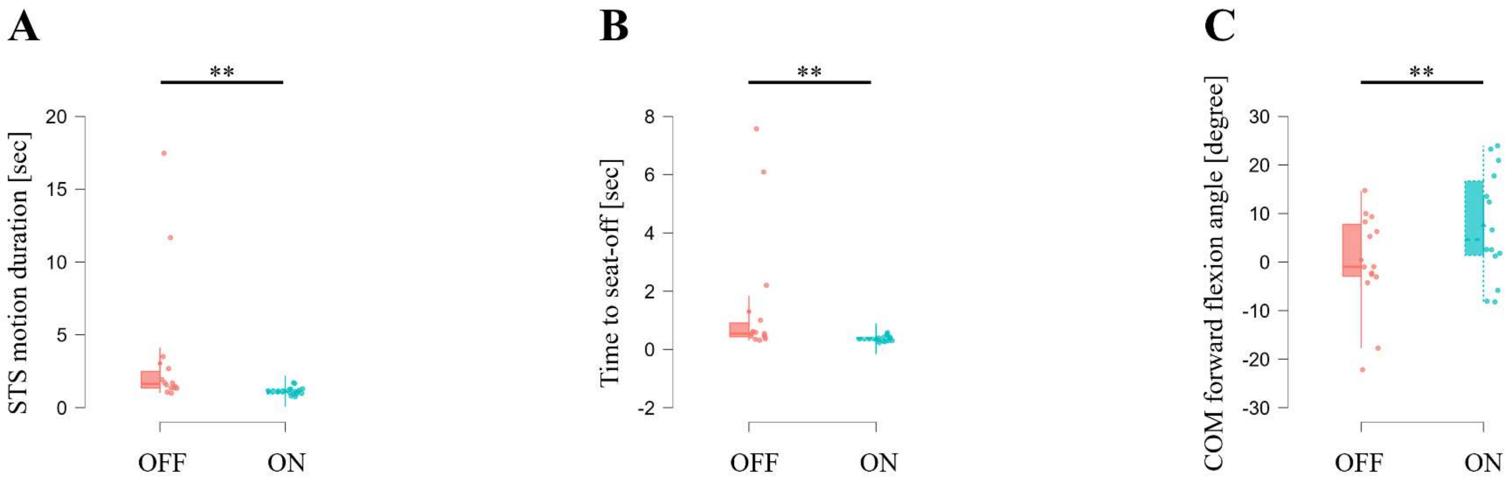

			95% CI	
state	Estimate	SE	Lower	Upper
OFF	3.046	0.513	2.040	4.052
ON	1.126	0.506	0.134	2.118

			95% CI	
state	Estimate	SE	Lower	Upper
OFF	1.303	0.253	0.807	1.799
ON	0.367	0.249	-0.122	0.855

			95% CI	
state	Estimate	SE	Lower	Upper
OFF	0.424	2.676	-4.822	5.670
ON	7.610	5.667	2.383	12.836

Figure S3: Comparison of the kinematic features between the OFF and ON states. (A) Mean sit-to-stand duration, (B) mean duration of each muscle synergy and (C) mean COM forward flexion angle. **$p < 0.01$.
