# Supplementary material for: Dopaminergic medication alters muscle synergy during sit-to-stand motion in Parkinson’s disease
Source: Front Neurol. 2026 Mar 23;17:1753476. doi: 10.3389/fneur.2026.1753476 (PMC13051405; doi:10.3389/fneur.2026.1753476)
Supplement: Supplementary file 2 [file Table_1.pdf]

Supplementary materials

Table S1: Detailed patient characteristics

ID	Age [years]	Disease duration [years]	MDS-UPDRS part III scores (OFF / ON)	Axial scores (OFF / ON)	Side and site of initial symptom onset	Levodopa-induced Dyskinesia	Hoehn and Yahr stage (OFF / ON)	Barthel index score	Levodopa Equivalent Daily Dose [mg]	Trimmed time before seat-off [s] (OFF / ON)	Trimmed time after seat-off [s] (OFF / ON)
1	60	8	15 / 6	6 / 0	right arm and leg	Yes	II / II	90	812.5	1 / 1	2 / 2
2	56	6	38 / 9	13 / 1	left hand	Yes	III / II	90	2070	1 / 1	2 / 2
3	71	8	42 / 10	14 / 3	four limbs	Yes	III / II	100	2040	1 / 1	2 / 2
4	63	10	54 / 18	20 / 3	left arm and leg	No	III / II	95	1165	7 / 1	7 / 2
5	74	8	44 / 16	18 / 5	left arm and leg	Yes	III / III	90	1799.25	1 / 1	2 / 2
6	67	14	48 / 14	18 / 2	right arm	Yes	II / II	95	900	1 / 1	2 / 2
7	68	7	61 / 17	13 / 1	right arm	Yes	II / II	85	750	2 / 1	3 / 2

Supplementary materials

8	62	6	26 / 8	21 / 2	right arm	Yes	II / II	100	1600	2 / 1	3 / 2
9	57	8	37 / 10	13 / 1	right arm	Yes	II / II	100	1215.4	1 / 1	2 / 2
10	63	12	44 / 19	13 / 7	left arm	Yes	III / III	55	1375	1 / 1	2 / 2
11	59	9	30 / 12	6 / 2	four limbs	Yes	II / II	90	1075	1 / 1	2 / 2
12	69	6	42 / 10	18 / 3	left leg	No	III / II	80	1650	10 / 1	15 / 2
13	50	14	38 / 12	11 / 1	right leg	No	II / II	100	2230	1 / 1	2 / 2
14	69	14	38 / 13	12 / 3	left hand	Yes	II / II	90	1650	3 / 1	3 / 2

Note. MDS-UPDRS = Movement Disorder Society-Unified Parkinson’s Disease Rating Scale. Axial score: the sum of MDS-UPDRS item 3.1 (speech), 3.2 (facial expression), 3.3 (neck), 3.9 (arising from chair), 3.10 (gait), 3.11 (FoG), 3.12 (posture stability), 3.13 (posture), and 3.14 (body bradykinesia).

Table S2: ANOVA summary table in Linear mixed model

**Muscle synergy features (Method)**	**Effect**	**F / ChiSq**	**p**	**p (bootstrap)**
Start time (Square root transformation + Parametric bootstrap LMM)	State	0.144	0.705	0.717
	Synergy	622.731	< .001	< .001
	State * Synergy	29.001	< .001	< .001
Duration time (Square root transformation + Parametric bootstrap LMM)	State	18.304	< .001	< .001
	Synergy	173.437	< .001	< .001
	State * Synergy	11.672	0.009	0.016
Average activation value (Standard LMM)	State	11.275	< .001	
	Synergy	198.29	< .001	
	State * Synergy	6.394	< .001	
Overlap time (Parametric bootstrap LMM)	State	15.133	< .001	< .001
	Synergy	263.152	< .001	< .001
	State * Synergy	6.044	0.049	0.046
COM horizontal position	State	4.144	0.042	0.033
	Synergy	655.644	< .001	< .001

(Square root transformation + Parametric bootstrap LMM)	State ✻ Synergy	25.469	< .001	< .001
COM vertical position (Parametric bootstrap LMM)	State	22.765	< .001	< .001
	Synergy	290.405	< .001	< .001
	State ✻ Synergy	8.433	0.038	0.035

Note. For variables analyzed with parametric bootstrap, the Chi-square statistic is reported instead of the F statistic, as the significance was determined through likelihood ratio testing with 1000 resamples.
